# A Case for Medical Discipline

**Published:** 1893-10-14

**Authors:** 


### A Case for Medical Discipline.

It may be within the remembrance of our readers that the case In *re* Macdonald, Sons, and Co. (Limited), where an application was made to the High Court to remove the names of several medical men from the list of contributories on the winding-up of this company, created a great deal of adverse comment in the medical and lay press. To enable the reader to follow the case, we summarise the following facts from the report which appeared in the *Times* of August 8th (vide *Times* Law Reports, No. 34, vol. ix., pages 643—645):—

The company was formed for the purpose of acquiring and carrying on the business of medicated wine manufacturers, which was purchased by the company from the two vendors. As part of the consideration there were to be paid to the vendors forty "founders' shares" of £25 each, to be allotted as fully-paid up to the persons named by the vendors as founders of the company. The company, with the consent, if not by direction of the vendors, entered into negotiation with various medical men to induce them to prescribe and recommend to their patients and tradesmen the goods dealt in by the company, promising to each doctor who undertook thus to recommend the company's wares a fully-paid 'founders' share. The negotiations were in some cases conducted directly between the doctors and the secretary of the company without mentioning the vendors.

In due course the Secretary filled in the names of the various doctors who were willing to accept founders' shares, including the applicants who brought the action referred to, and certificates of shares were sent to them. The Secretary wrote to the doctors that the founders' shares were fully paid, and the recipients would incur no liability by acceptance.

Owing to certain irregularities committed by the company, the liquidator held that the recipients of the founders' shares were liable to pay the full amount of £25, and the action in question was brought with the view of avoiding the payment by the doctors of the £25 claimed upon each founders' share. The legal aspects of the case are of no interest to our readers, but we may add that in the end the Court held that the doctors were not liable to pay anything upon the founders' shares. The judge, however, Mr. Justice Vaughan Williams, in the course of his judgment, stated that "he could not part with the case without saying that it was a sad thing that members of a learned profession should have consented to take shares on these terms. It might be that the individual doctors thought well of the wares of this company, and that in praising them they acted as their consciences dictated, but to take shares in consideration of praising the wares of the company was very much like taking them as bribes, and it was impossible not to have a strong feeling that if those who took the shares under such circumstances were held liable as contributories, it would serve them right." His Lordship refused to give the applicants costs as against the official receiver.

In view of the remarks made by the Judge, it is perfectly clear that the corporations who granted diplomas to the medical practitioners in question cannot let the matter rest where it stands without fuller and further investigation. In the interests of the whole medical profession and the public, we feel it to be our duty to publish the names of those who made the application to the Court by way of summons as holders of the founders' shares.

The Order of the Court, which was dated August 7th, 1893, runs as follows:—

"Upon application by summons dated June 5th, 1893, of

Alfred Phillips, of 22, Petherton Road, Highbury, in the county of Middlesex, M.R.C.S., L.S.A.

William Charles Worley, of 103, Green Lanes, Highbury, aforesaid, M.R.C.S.

H. George Sworn, of 5, Highbury Crescent, Highbury, aforesaid, M.R.C.S.

William Blagdon Richards, of 329, Goswell Road, in the same county, L.S.A.

Alfred F. Richards, of Castle House, Carmarthen, M.B., B.C.

Robert J. Carter, of 4, St. John's Wood Terrace, in the county of Middlesex, M.B., M.R.C.S.

Dr. Hoare, of Clifton House, Aston Road, Birmingham.

R. S. Cundell, of Brunswick House, Kew, M.D.

F. Arthur Farr, of 116, Earl's Court Road, Kensington, in the county of Middlesex, M.D."

All the names will be found in the Medical Register for 1893, though in some cases some of the qualifications are not given; the address in one or two cases differs; and in one case one of the initials appears to be wrong. Pending action by the universities and colleges immediately concerned, we purposely refrain from further comment.

